# Editorial: How the COVID-19 security measures have influenced the psychological therapies procedures and therapeutic elements

**DOI:** 10.3389/fpsyg.2023.1151565

**Published:** 2023-02-22

**Authors:** Eleni Petkari, Domenico Giacco, Ana Calvo

**Affiliations:** ^1^School of Health Sciences, Universidad Internacional de La Rioja, Logroño, Spain; ^2^Warwick Medical School, University of Warwick, Coventry, United Kingdom; ^3^Departamento de Personalidad, Evaluación y Psicología Clínica, Facultad de Psicología, Universidad Complutense de Madrid, Madrid, Spain

**Keywords:** teletherapy, COVID-19, psychotherapy, mask-wearing, online therapeutic settings

The COVID-19 pandemic brought major changes in mental health services. Clinicians and service users had to face challenges related to the restrictions for attending appointments, as well as the physical distancing and mask-wearing practices during the less frequent face-to-face therapeutic sessions. To achieve service continuity in mental health care, services had to rapidly shift to a new form of service delivery, transitioning from purely face-to-face to online, or hybrid modalities. Teletherapy, understood as the application of psychological interventions using an online format (Joint Task Force for the Development of Telepsychology Guidelines for Psychologists, 2013) was the most frequent modality used across services. Teletherapy has showed good acceptability by both clinicians and people with mental health difficulties (Appleton et al., 2021), and treatment effectiveness (Poletti et al., 2021). Although there are several challenges related to the lack of clear guidelines for its implementation (Smith et al., 2020), the unprecedented need for teletherapy use during the COVID-19 pandemic offered a unique opportunity for in-depth research of its applications across the globe, in a variety of clinical settings (Torous et al., 2020).

This collection includes studies on experiences of teletherapy during the COVID-19 pandemic. It aims to help shape best practices for its use in clinical practice, and to understand how restrictions of physical distancing and mask-wearing may have influenced face-to-face clinical practice. The collection includes three original papers, one review, and one meta-analysis, which offer a comprehensive overview of practices of psychotherapy during the COVID-19 pandemic.

Two of the articles focused on the clinicians' views and challenges when delivering psychological therapies online. Békés et al. carried out a longitudinal assessment with a large sample of psychotherapists, mainly from North America. Their aim was to examine how clinicians perceived the psychotherapy transition from face-to-face to the online environment at the beginning of the pandemic, and whether their perceptions changed 3 months later. Their findings revealed that the clinicians perceived challenges for: establishing emotional connection with the patients, avoiding distractions, preserving confidentiality, and establishing professional boundaries. Importantly, baseline levels of the challenges related to emotional connection and confidentiality seemed to predict more negative attitudes toward online therapy, as well as lower perceived therapeutic efficacy 3 months later.

Similarly, Mancinelli et al. examined the self-perceptions of psychotherapists using online environments during the first months of the pandemic in Italy. Psychotherapists seemed to perceive themselves as confident and competent for implementing teletherapy. They also reported a therapeutic style change, perceiving themselves as more talkative and directive compared to the pre-pandemic era. However, psychotherapists also felt that this novel form of service provision caused them more fatigue, possibly due to the limited access to non-verbal cues during the teletherapy sessions, which required them to put more effort on collecting information from the clients' background.

Another study also focused on the impact of limited access to non-verbal cues, describing the experience of face-to-face therapy during the period of restrictions due to COVID-19. Specifically, Ribeiro et al. conducted a study with psychotherapists in Portugal after two lockdown periods and aimed to examine the clinicians' experience of wearing a mask during the face-to-face therapeutic sessions, and the perceived impact of mask-wearing on the therapeutic procedure. As in the previous study, clinicians mentioned that their experience of mask-wearing was causing them fatigue, due to the effort they had to put on detecting non-verbal cues. However, they also felt that mask-wearing did not affect the therapeutic alliance, or any other aspects of the process. Importantly, clinicians also felt that face-to-face sessions, albeit the use of mask, were necessary to overcome the disadvantages of online therapy and adhere to the clients' preferences for interaction. Overall, participants in this study did not perceive a negative impact of mask-wearing on the therapeutic experience, perhaps because the study was carried out after two lockdown periods, and therefore a habituation process may have taken place.

A scoping review focused on the implementation of drama therapy in the online environment. Shi and Jing offer an overview of the advantages and disadvantages of applying dramatherapy through online environments. The authors conclude that the online experience of dramatherapy seems to instigate creativity, offer opportunities for insight and for broadening the therapeutic space to include more intimate settings, and offer flexible therapy sessions-scheduling. On the other hand, the review reports the limitations depicting confidentiality concerns and lack of engagement due to the screen barriers. The authors suggest that overcoming these obstacles may contribute to the establishment of drama therapy as an equally attractive teletherapy approach, compared to more traditional approaches.

Finally, a meta-analysis offered a systematic examination of the effectiveness of online therapy for people suffering from mental health difficulties due to the COVID-19 restrictions. Chi et al. based their findings on 13 Randomized Controlled Trials and revealed that the interventions, mostly based on Cognitive Behavioral Therapy and mindfulness, were effective for reducing depression, anxiety, and stress levels, but not for improving sleep quality. Also, interventions implemented by a clinician were more effective for tackling anxiety, but not depression or stress, compared to self-help online applications.

As suggested by the articles included in this collection, during the COVID pandemic clinicians had to face a series of challenges derived by the constraints dictated by governmental directives on physical distancing. However, there is evidence that tele-therapy has been beneficial for people with common mental disorders, on which these studies have focused, and one can argue that it effectively contributed to guaranteeing service continuation. Similarly, clinicians overcame challenges related to wearing masks during sessions, to ensure service delivery standards were fulfilled. The findings of this collection offer valuable perspectives of the clinicians' practice, however the question on how this sudden and mandated change to practice may have affected the service users' therapeutic experience remained unanswered by the included studies. Currently, restrictions are relaxed across the world, therefore it is still to be established whether the adaptations made during the COVID pandemic will result in a paradigm shift in service delivery toward increasing the use of teletherapy.

## Author contributions

EP wrote the first version of the editorial. All authors edited and contributed to the final version.
